# Appropriately adapted properties of hot-extruded Zn–0.5Cu–xFe alloys aimed for biodegradable guided bone regeneration membrane application

**DOI:** 10.1016/j.bioactmat.2020.09.019

**Published:** 2020-10-09

**Authors:** Wentai Zhang, Ping Li, Gang Shen, Xiaoshan Mo, Chao Zhou, Dorothea Alexander, Frank Rupp, Jürgen Geis-Gerstorfer, Haijun Zhang, Guojiang Wan

**Affiliations:** a Key Laboratory of Advanced Technologies of Materials, Ministry of Education, School of Materials Science and Engineering, Southwest Jiaotong University, Chengdu, 610031, China; b Section Medical Materials Science and Technology, University Hospital Tübingen, Osianderstrasse 2-8, Tübingen, 72076, Germany; c Beijing Advanced Innovation Center for Materials Genome Engineering, School of Materials Science and Engineering, University of Science and Technology Beijing, Beijing, 100083, China; d Department of Oral and Maxillofacial Surgery, University Hospital Tübingen, Osianderstrasse 2-8, Tübingen, 72076, Germany; e Department of Interventional and Vascular Surgery, The Tenth People's Hospital of Shanghai, Tongji University, Shanghai, 200072, China; f National United Engineering Laboratory for Biomedical Material Modification, Branden Industrial Park, Qihe Economic & Development Zone, Dezhou, Shandong, 251100, China

**Keywords:** Zn-based biodegradable metal, Mechanical properties, Degradation behavior, Biocompatibility, Guided bone regeneration membrane

## Abstract

Appropriately adapted comprehensive mechanical properties, degradation behavior and biocompatibility are prerequisites for the application of Zn-based biodegradable implants. In this study, hot-extruded Zn–0.5Cu–xFe (x = 0.1, 0.2 and 0.4 wt%) alloys were fabricated as candidates for biodegradable materials for guided bone regeneration (GBR) membranes. The hot-extrusion process and Cu alloying were expected mostly to enhance the mechanical properties, and the Fe alloying was added mainly for regulating the degradation. The microstructure, mechanical properties and *in vitro* degradation behavior were systematically investigated. The ZnCuFe alloys were composed of a Zn matrix and FeZn_13_ phase. With increasing Fe content, a higher FeZn_13_ phase precipitation with larger particles was observed. Since elongation declined significantly until fracture with increasing Fe content up to 0.4 wt%, the ZnCuFe (0.2 wt%) alloy achieved a good balance between mechanical strength and ductility, with an ultimate tensile strength of 202.3 MPa and elongation at fracture of 41.2%. Moreover, the addition of Fe successfully accelerated the degradation of ZnCuFe alloys. The ZnCuFe (0.2 wt%) alloy showed relatively uniform corrosion in the long-term degradation test. Furthermore, extracts of the ZnCuFe (0.2 wt%) alloy showed no apparent cytotoxic effects against L929 fibroblasts, Saos-2 osteoblasts or TAg periosteal cells. The ZnCuFe (0.2 wt%) alloy exhibited the potential to inhibit bacterial adhesion of *Streptococcus gordonii* and mixed oral bacteria. Our study provides evidence that the ZnCuFe (0.2 wt%) alloy can represent a promising material for the application as a suitable GBR membrane.

## Highlights

• Hot-extruded Zn-0.5Cu-xFe alloys designed for biodegradable GBR membrane application.
• The Zn–0.5Cu–0.2Fe alloy showed a good balance between tensile strength and ductility.
• The degradation behavior was improved when the Fe content is less than 0.4 wt%.
• The Zn–0.5Cu–0.2Fe alloy exhibited good cytocompatibility and antibacterial ability.
• This Zn-based alloy provide appropriately adapted properties for degradable GBR membrane.

## Graphical abstract

**Figure:**
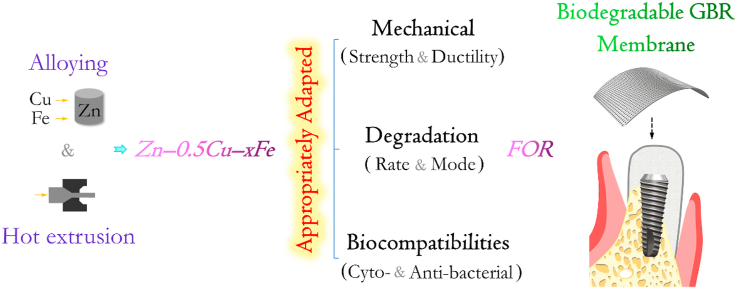

## 1 Introduction

Guided bone regeneration (GBR) is an effective approach for bone tissue augmentation to address insufficient bone volume around dental implants [[1], [2], [3]]. In principle, a GBR membrane not only provides stable space for new bone formation but prevents infiltration of surrounding epithelium and connective tissue as well [4,5]. Thereby, the ideal GBR membrane includes the following main characteristics: maintenance of space-making ability, proper degradability, excellent biocompatibility, adequate cell-occlusiveness and clinical feasibility [4,6]. Commercial GBR membranes can be mainly classified as biodegradable and non-biodegradable membranes [6,7]. To date, biodegradable membranes have been widely used for GBR treatment; they mainly include natural polymers, synthetic polymers and their blends [8]. The inherent shortcoming in the utilization of these membranes is the poor space-making ability due to their limited mechanical properties [4,5]. This might lead to collapse of the membrane and, finally, the impairment of new bone formation. In contrast, a typical bioinert non-biodegradable membrane, such as a titanium (Ti) membrane, can provide effective space-making ability for the bone reconstruction of maxillofacial defects. Nevertheless, the limitation of non-biodegradable membranes is the additionally required surgery for their removal after new bone formation. Therefore, alternative materials such as biodegradable metallic GBR membranes are needed.

Biodegradable metals (absorbable metals), such as magnesium (Mg), iron (Fe), zinc (Zn) and their alloys, have been considered as revolutionary metallic biomaterials due to their biodegradability, good biocompatibility and mechanical properties [[9], [10], [11]]. Among them, Zn and Zn-based alloys have recently been considered as the leading candidate materials for biodegradable implants applications [[12], [13], [14], [15]]. Zn-based composites and porous scaffolds have also been designed for regenerative purposes in tissue engineering [16,17]. On one hand, elemental zinc is one of the essential nutrients in the human body, where it influences and participates in various physiological processes [18,19]. On the other hand, Zn and its alloys exhibit a moderate degradation rate, compared to Fe and Mg, and the degradation products released do not contain hydrogen [9,20]. More importantly, the potential osteoinductive ability of Zn and Zn-based materials has been demonstrated [[21], [22], [23]]. Nevertheless, the limitation of as-cast pure zinc is its insufficient mechanical properties concerning most medical implants, i.e. low strength (ultimate tensile strength approx. 30 MPa) and ductility (elongation < 0.25%) [24]. Considering the GBR membrane application, the mechanical strength and ductility are both important for space-maintaining and specific geometry remodeling, respectively [6]. Moreover, the degradation process could lead to a higher risk of premature failure and therefore more demanding mechanical requirements as compared to the non-degradable counterparts. To improve the comprehensive mechanical properties of zinc, alloying and processing treatments have been proven to be an effective way [[25], [26], [27]].

For alloying elements, copper (Cu) has been proposed as a promising choice for Zn alloys, because of the significant enhancement for both strength and ductility of the Zn matrix, its bio-safety as well as even potential antibacterial function [28,29]. However, fibroblasts are extremely sensitive to the Cu^2+^ concentration. The half-maximal inhibitory concentration of Cu^2+^ to murine fibroblasts (L929) is 41.5 μM, much lower than that of Zn^2+^ (92.8 μM) [30]. Furthermore, it has been reported that 100% extracts of Zn–4Cu alloy inhibit the proliferation of mouse fibroblasts [12], implying the potential risk of high content of Cu alloying element. On the other hand, it has been reported that Zn–0.5 wt%Cu showed an almost twofold increase in yield strength (YS) and ultimate tensile strength (UTS), and an increase in elongation at fracture to 18% compared to Zn [31]. So, in the present work 0.5 wt% Cu was selected, which was supposed to be sufficient for the mechanical improvement as well as maintaining a low content level. The other alloying element was for regulating the degradation rate of the Zn–Cu alloys as another important concern. Considering the typical *in vitro* degradation rates of Zn–Cu alloys (< 150 μm/year), a typical 100-200 μm-thick metallic GBR membrane would take about one year to degrade completely [12,28]. It is therefore necessary to accelerate the degradation rate of Zn–Cu alloys to match the bone healing process. Iron (Fe) is an essential element for the human body and necessary for the functionality of enzymes and oxygen transportation [32]. Importantly, alloying with Fe induces a higher Zn corrosion rate *in vivo* due to galvanic corrosion between the secondary phase formed and the Zn substrate [33]. In addition, L929 fibroblasts show a much higher tolerance to Fe^2+^ and Fe^3+^ (< 6.95 mM and 5.42 mM, respectively) than to Cu^2+^ and Zn^2+^ [30]. It seems that Fe is a suitable ternary alloying element for Zn–Cu-based alloys for GBR membranes. However, the content of Fe needs to be further investigated because remarkably reduced elongation at fracture has been observed when the Fe concentration exceeds 0.5 wt% [34]. Thus, the Fe contents less than 0.5 wt% were selected in this study to accelerate the degradation while maintaining adequate ductility. We fabricated a series of Zn–0.5Cu–xFe (x = 0.1, 0.2 and 0.4 wt%) alloys to achieve the optimal balanced mechanical properties and degradation behavior. Moreover, hot extrusion has been reportedly applied to further enhance mechanical properties of Zn alloys whereby it can refine readily the grains of Zn matrix [28,35]. In addition, the refined grains could bring about more uniform corrosion on Zn alloys, which is beneficial for maintaining the mechanical integrity during implantation [36].

**Scheme 1:**
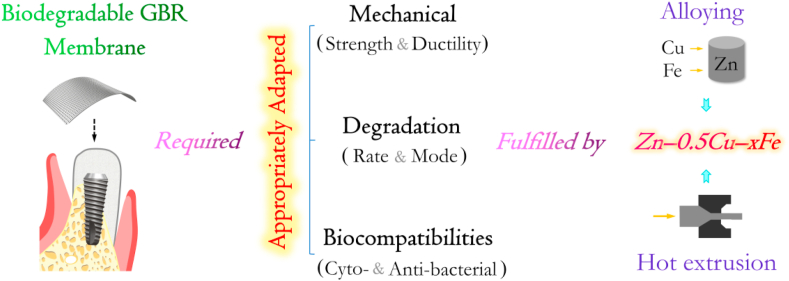
Schematic of strategy to fabricate hot-extruded Zn–0.5Cu–xFe alloys with appropriately adapted mechanical, degradation and biocompatible properties for GBR membranes.

The challenging objectives were to produce a biodegradable material with sufficient mechanical properties, proper degradation behavior, cytocompatibility and antibacterial properties with the overall potential for use as GBR membranes, as illustrated in Scheme 1. Based on these study aims, the microstructure and tensile properties were investigated. To mimic clinical conditions, the degradation behavior was determined in different simulated body fluids, namely cell culture medium and artificial saliva. Additionally, the cytotoxicity and antibacterial properties of the finally selected alloy were evaluated from the perspective of GBR membrane application.

## 2 Materials and methods

### 2.1 Material preparation

Zn–0.5%Cu–xFe (x = 0, 0.1, 0.2 and 0.4 wt% (denoted as ZnCu, ZnCuFe (0.1), ZnCuFe (0.2) and ZnCuFe (0.4), respectively)) were prepared with high-purity Zn ingot (99.99 wt%), Cu wire (99.9 wt%) and Fe wire (99.99 wt%), provided by Branden Medical Devices Co., Ltd, China. The wires of Cu and Fe were chosen in order to shorten the melting time. Melting was performed in an electric resistance furnace at 650 °C. Afterwards, the melt was gravity-cast into cylindrical cast-iron molds and cooled down in the air. The ingots obtained, with a diameter of 60 mm and thickness of 30 mm, were subsequently heated to 350 °C for 30 min for homogenization. Finally, the as-cast alloys and pure Zn were extruded at 300 °C with an extrusion ratio of 22 : 1 and a rate of 0.4 mm/s. Samples for the characterization and degradation measurements were cut into disks with 10 mm diameter and 1.5 mm thickness. For the tensile tests, the samples were machined into a dog-bone shape parallel to the extrusion direction according to ASTM E8/E8M-16a [37]. For the cytotoxicity and antibacterial tests, plates of 30 mm × 10 mm × 1.5 mm and of 7 mm × 7 mm × 1.5 mm, respectively, were prepared. All the samples were polished with silicon carbide grinding paper up to 2000 grit and then ultrasonically cleaned in ethanol and deionized water for 10 min, respectively. Samples for the observation of microstructure were further polished with W1.0 diamond abrading agent to a mirror surface, and then ultrasonically cleaned by ethanol for 10 min. Prior to the immersion test and biological tests, specimens were disinfected using ultraviolet radiation with a wavelength of 254 nm for 1 h.

### 2.2 Microstructure characterization and mechanical tests

The microstructures of the samples were observed using a tridimensional microscope (Motic Images Plus 2.0 ML). All samples were etched in a solution consisting of 50 g/L CrO_3_, 15 g/L Na_2_SO_4_ and deionized water before observation. The second phases and composition were further determined using a field-emission scanning electron microscope (FE-SEM, JSM-7401F, JEOL, Japan) and a coupled energy dispersive spectroscope (EDS). X-ray diffraction (XRD, X'Pert Philips) was applied to characterize the phase composition with a CuK-alpha radiation source. The glancing angle was set to 2° in a 2θ range from 20° to 90° with a step size of 0.25°. The mechanical tensile properties were evaluated using an universal loading machine (LabTest Instron-5567) with a deformation rate of 1 mm/min at room temperature. Three samples of each group were tested. The yield strength (YS) and ultimate tensile strength (UTS) were obtained from the stress-strain curves. Elongation at fracture was calculated by measuring the gauge length of samples before and after the tensile test.

### 2.3 In vitro degradation measurements

Electrochemical measurements were performed to evaluate the degradation behavior of samples on an electrochemical workstation (IM6, Zahner, Germany) at 37 ± 0.5 °C. Three electrodes were used for the measurements: a saturated calomel reference electrode, a platinum counter electrode (1.5 cm × 1.5 cm) and a working electrode (tested sample, 0.79 cm^2^ exposure area). The back of all samples was polished carefully to remove the oxides and electrically connected with copper wire. Then, the samples were sealed with silicone rubber to expose only the front surface. To simulate the submucosa and intraoral environment, α-modified minimum essential medium (α-MEM, Hyclone, Logan, UT, USA) and modified Fusayama–Meyer artificial saliva were chosen to be the electrolytes for the degradation tests. The artificial saliva was composed of 0.4 g/L NaCl, 0.4 g/L KCl, 0.795 g/L CaCl_2_·2H_2_O, 0.69 g/L NaH_2_PO_4_·H_2_O, 0.005 g/L Na_2_S·9H_2_O, 0.3 g/L KSCN, 1 g/L carbamide (urea) and deionized water with a pH value of 6 [38]. The potentiodynamic polarization (PDP) curves were recorded with a scanning rate of 1 mV/s. The Tafel method was used to calculate the free current density (*i*_corr_) by extrapolation from the linear cathodic polarization zone, about 60 mV lower than the open-circuit potential. Electrochemical impedance spectroscopy (EIS) was measured with a sinusoidal perturbating signal of 10 mV around the open-circuit potential in a Faraday cage. The perturbating frequency ranged from 200 kHz to 0.1 Hz with four points per frequency decade. The obtained EIS data were fitted with ZSimpWin (version 3.60). The electrochemical measurements were repeated at least five times for each sample for statistical purposes.

To further investigate the long-term degradation behavior, an immersion test was performed for 21 days. Samples were immersed in α-MEM medium and modified Fusayama-Meyer artificial saliva under cell culture conditions (5% CO_2_, 95% humidity, 37 °C) with a ratio of surface area to solution volume of 1.25 cm^2^/mL, which is commonly used for evaluating the degradation of biodegradable metallic GBR membranes to simulate its physiological environment in craniomaxillofacial area [39,40]. The electrolytes were refreshed every 3 days to mimic the fluid exchange at implantation sites. After 21 days of immersion, samples were taken out and cleaned carefully with deionized water. The surface morphology and composition of corrosion products were examined by FE-SEM and coupled EDS. The corrosion underneath the surface layer was also observed by removing the corrosion products according to ASTM G1-03(2017)e1 [41]. Briefly, the samples were immersed in 200 mg/mL CrO_3_ for 1 min at 80 °C and then cleaned three times using distilled water. The weight of samples before immersion and after removing the corrosion products was measured to calculate the corrosion rate (*CR*), namely the degradation rate, in μm/year as following [42]:

(1) CR=(K×W)/(A×T×D)

where K is a constant (8.76 × 10^7^); W is the weight loss in g; A is the surface area exposed to the electrolyte in cm^2^; T is the immersion time in hours and D is the density in g/cm^3^.

### 2.4 Cytotoxicity test

An indirect cytotoxicity test was performed to evaluate the finally selected ZnCuFe alloy by the sample extract assay according to ISO 10993-5: 2009 [43]. Three types of cell lines were used, namely mouse fibroblasts (L929), human osteosarcoma cells (Saos-2), and human immortalized cranial periosteal cells (TAg). The sample extracts were prepared using the respective cell culture media without serum with an extraction ratio of 1.25 cm^2^/mL for 24 h under cell culture conditions, according to ISO 10993-12: 2012 [44]. The metallic ion concentrations of the extracts were measured using an inductively coupled plasma optical emission spectrometer (ICP-OES; Optima 4300DV, Germany), and pH values were also detected. In addition, sample extracts were supplemented with the appropriate quantity of serum prior to testing.

Cell viability was visualized qualitatively by live/dead staining (Sigma-Aldrich, Taufkirchen, Germany). Cells were seeded into 12-well plates at a seeding density of 3 × 10^4^ cells/cm^2^ overnight. Then, the media were exchanged for the sample extracts, with Ti–6Al–4V extracts as a negative control and Cu extracts as a positive control. Cells were stained by live/dead staining reagent, containing 1.25 μg/mL ethidium bromide (EB) and 25 μg/mL fluorescein diacetate (FDA) in Hank's balanced salt solution (HBSS), according to our previous protocol [45]. Cell viability was assessed using a fluorescence microscope (Optiphot-2, Nikon, Tokyo, Japan). Additionally, relative cell metabolic and proliferation activities were quantitatively evaluated by the cell counting kit-8 assay (CCK-8, Dojindo, Kumamoto, Japan) and the bromodeoxyuridine assay (BrdU, Roche, Mannheim, Germany), respectively. Briefly, cells were cultured in 96-well plates at a seeding density of 3 × 10^4^ cells/cm^2^ and incubated overnight. Then, cells were exposed to the corresponding sample extracts with BrdU labeling for 24 h. After that, CCK-8 reagent was added for 2 h, and the spectrophotometric absorbance at 450 nm was measured by a microplate reader (Infinite F50, Tecan, Grödig, Austria). The potential inhibition of relative metabolic activity (*RMA*) was calculated as following:

(2) $$RMA\left(\%\right)=\left(\left(OD_{\text{test}}-OD_{\text{blank}}\right)/\left(OD_{\text{negative}}-OD_{\text{blank}}\right)\right)\times 100\%$$

where $OD_{\text{test}}$ is the mean optical density (OD) value of the test groups; $OD_{\text{negative}}$ is the mean OD value of the negative control and $OD_{\text{blank}}$ is the mean OD value of the cell culture media with the CCK-8 reagent, as previously reported in detail [45]. After fixation, cells were then incubated with an anti-BrdU-POD working solution, and optical densities were subsequently quantified at 492 nm (reference wavelength 620 nm) according to the manufacturer's instructions.

### 2.5 Antibacterial properties test

The antibacterial properties of the tested ZnCuFe alloy were determined by bacterial adhesion on the surface, with pure Zn as a material reference. Ti–6Al–4V and pure Cu were used as controls. Considering the later GBR application, the *Streptococcus gordonii* strain DL1 (*S. gordonii*) and mixed oral bacteria were used. These species, mainly involved in postoperative infections after transoral surgeries, are proposed to reflect the oral environment [[46], [47], [48]]. Furthermore, *S. gordonii* and mixed oral bacteria were incubated and collected, as we previously reported in detail [46,49]. Briefly, mixed oral bacteria were freshly collected from a periodontally healthy volunteer after obtaining informed consent. Then, *S. gordonii* and mixed oral bacteria were respectively grown in Schaedler medium (Beckton Dickinson, Heidelberg, Germany) overnight at 37 °C. Afterwards, 3 mL suspensions of both bacteria, i.e., the *S. gordoni*i suspension and the mixed oral bacteria suspension, were added to the sample surfaces in the 12-well plates, respectively. After incubation for 12 h, the surfaces were gently rinsed with HBSS (Boichrom, Berlin, Germany). The bacteria adhering to the surface were stained using live/dead staining (L13152, Invitrogen, USA), followed by observation using a fluorescence microscope.

To quantitatively evaluate the antibacterial properties of the ZnCuFe alloy, the antibacterial rate for planktonic bacteria was measured, in accordance with the protocol as reported before [50,51]. Specifically, *S. gordonii* and mixed oral bacteria were cultured in Schaedler medium and incubated overnight at 37 °C. Bacteria concentration was quantified by absorption spectroscopy at a wavelength of 620 nm. Bacteria suspension was adjusted to OD value 0.54 at 620 nm, approximately equivalent to 1 × 10^8^ bacteria mL^−1^ [46]. Furthermore, three samples per group were inoculated with 3 mL diluted bacteria suspension (1 × 10^6^ bacteria mL^−1^) in 12-well plates for 12 h at 37 °C and 120 rpm. Bacteria suspensions without samples were used as controls, and Ti–6Al–4V and pure Zn were used as material references. After 12 h of incubation, the OD value was measured at 620 nm. To calculate the antibacterial rate (*AR*) for planktonic bacteria (%), the following formula was used:

(3) $$AR\left(\%\right)=\left(OD_{\text{control}}-OD_{\text{sample}}\right)/OD_{\text{control}}\times 100\%$$

where $OD_{\text{control}}$ is the mean OD value of the control; $OD_{\text{sample}}$ is the mean OD value of the tested sample [50,51]. The pH value of the respective bacteria media was recorded. Afterwards, six specimens of bacterial media per group were collected and then centrifuged at 1400 rpm for 15 min. The respective 2 mL supernatant was withdrawn and further filtered by a 0.2 μm filter. ICP-OES measurements were performed to detect the metallic ion concentration in the respective bacterial media. Three independent experiments were carried out to ensure the reproducibility.

### 2.6 Statistical analysis

The experimental data were expressed as mean and corresponding standard deviations. One-way analysis of variance (ANOVA) followed by post hoc Tukey's multiple comparison tests was applied to evaluate the statistical significance of differences between groups. Statistical analyses were performed using GraphPad PRISM version 6.1 (GraphPad Software, Inc., San Diego, US). A P-value < 0.05 was accepted as statistically significant.

## 3 Results

### 3.1 Microstructural characterization and mechanical properties

**Fig. 1:**
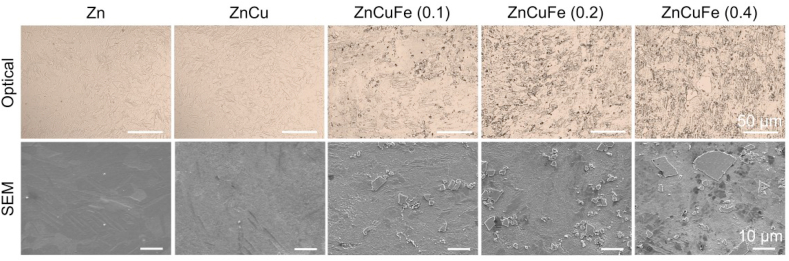
Optical (upper panel) and SEM (lower panel) images of hot-extruded Zn, ZnCu, ZnCuFe (0.1 wt%), ZnCuFe (0.2 wt%) and ZnCuFe (0.4 wt%) alloys.

Fig. 1 shows the microstructures of hot-extruded Zn, ZnCu and ZnCuFe alloys examined by optical microscopy and SEM. As illustrated, the microstructure of ZnCu alloy was similar to that of pure Zn. No apparent secondary phase was observed in the Zn matrix. After alloying with Fe, distinct solidification microstructures could be detected. Second-phase particles in the Zn matrix were recognized, with higher abundance when Fe content increased. Among alloys, ZnCuFe (0.2 wt%) showed a relatively well-distributed secondary phase. The size of the secondary phase formed on ZnCuFe (0.2 wt%) alloy was relatively uniform, whereas some second-phase particles with diameters of tens of microns were found when the Fe content was increased up to 0.4 wt%.

**Fig. 2:**
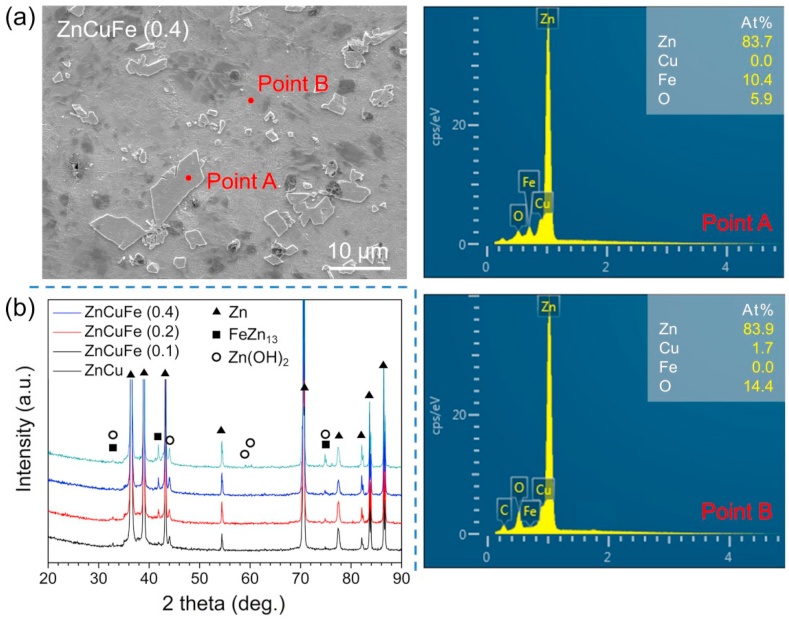
Microstructure analysis of the hot-extruded ZnCu and ZnCuFe alloys. (a) EDS results corresponding to the secondary phase and Zn matrix marked by red points on the ZnCuFe (0.4 wt%) alloy surface. (b) XRD patterns and phase characterization of the hot-extruded ZnCu, ZnCuFe (0.1 wt%), ZnCuFe (0.2 wt%) and ZnCuFe (0.4 wt%) alloys; The phase Zn (reference card 00-004-0831), FeZn_13_ (reference card 03-065-1238) and Zn(OH)_2_ (reference card 00-041-1359) were identified.

The chemical composition of the second phase and Zn matrix of the ZnCuFe (0.4 wt%) alloy are each marked by points A and B in Fig. 2a. The EDS results reveal that the secondary phase was mainly composed of Zn, Fe and O elements, whereas a small amount of Cu was detected in the Zn matrix apart from Zn, C and O elements. XRD spectroscopy was performed to identify the secondary phase (Fig. 2b). According to the XRD analysis, the ZnCuFe alloys were principally composed of FeZn_13_ and Zn matrix. Thus, the Fe-containing secondary phase can be identified as FeZn_13_. Further, the intensity of the FeZn_13_ peak increased significantly with increasing Fe content, which is in agreement with the observation of the microstructure. Note that weak peaks of Zn(OH)_2_ can be detected on the samples, which should come from the naturally formed hydroxide on the alloy surface when exposed to the atmosphere. It is noteworthy that the Cu-containing secondary phase was not spotted by EDS and XRD analysis.

**Fig. 3:**
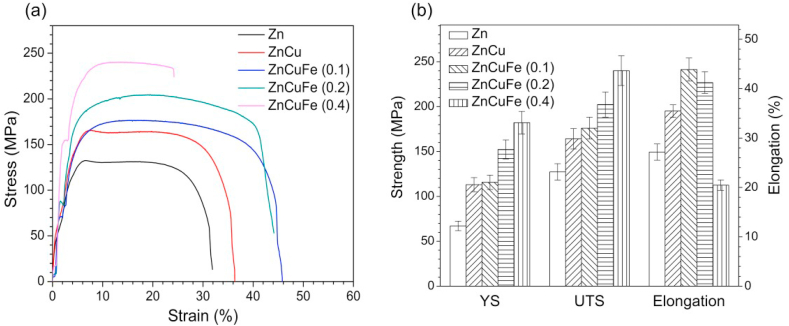
Mechanical performance of the hot-extruded Zn and Zn-based alloys. (a) Representative stress-strain curves and (b) calculated yield strength (YS, MPa), ultimate tensile strength (UTS, MPa), and elongation at fracture (%).

Fig. 3 shows the tensile mechanical properties of hot-extruded Zn, ZnCu and ZnCuFe alloys. The YS and UTS values for Zn were the lowest among all the samples (67.1 and 127.3 MPa, respectively). After alloying with 0.5 wt% Cu, the YS and UTS values increased significantly, up to 113.1 and 164.2 MPa, respectively. Also, ZnCuFe (0.1 wt%) alloy exhibited a tensile strength (115.7 MPa for YS, 176.0 MPa for UTS) comparable to that of ZnCu alloy. Furthermore, the YS and UTS values were both enhanced with an increase in Fe content (152.3 and 202.3 MPa, respectively, for ZnCuFe (0.2 wt%), and 182.1 and 240.1 MPa, respectively, for ZnCuFe (0.4 wt%). Notably, elongation at fracture declined significantly when the Fe concentration exceeded 0.2 wt%. Specifically, ZnCuFe (0.4 wt%) alloy showed the lowest elongation at fracture (20.5%) among the samples. The elongation at fracture of ZnCuFe (0.2 wt%) was 41.2%, being slightly lower than that of ZnCuFe (0.1 wt%) (43.9%), but still much higher than that of Zn and ZnCu alloy with 27.2% and 35.5%, respectively.

### 3.2 Degradation behavior in simulated body fluid and artificial saliva

**Fig. 4:**
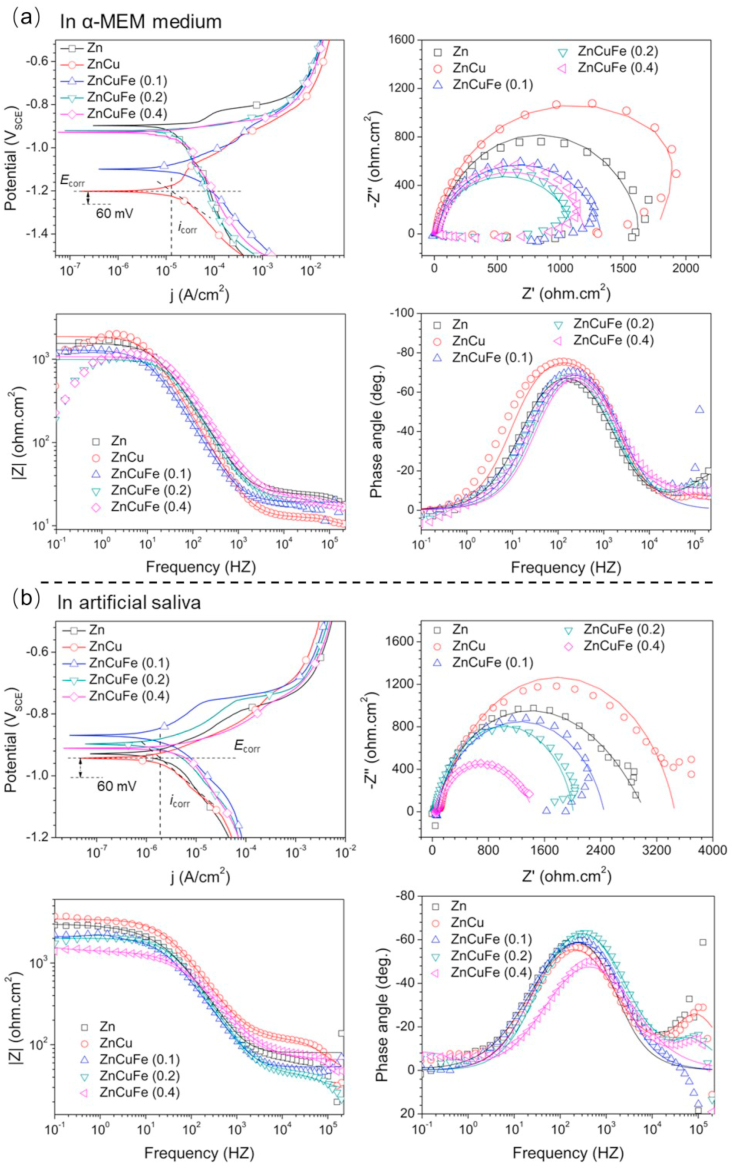
Potentiodynamic polarization (PDP) curves and electrochemical impedance spectra (EIS) of the hot-extruded Zn, ZnCu, ZnCuFe (0.1 wt%), ZnCuFe (0.2 wt%) and ZnCuFe (0.4 wt%) alloys in (a) α-MEM and (b) artificial saliva at 37 ± 0.5 °C. The measured EIS data and the fitting results are presented as dotted and solid lines, respectively.

The electrochemical corrosion results of the samples in α-MEM and artificial saliva at 37 ± 0.5 °C are illustrated in Fig. 4. The calculation and fitting results are listed in detail in Table 1. For the PDP measurements, ZnCu alloy exhibited the lowest *i*_corr_ value (10.68 μA/cm^2^ in α-MEM, 1.99 μA/cm^2^ in artificial saliva) among all the samples in both α-MEM and artificial saliva, indicating the excellent corrosion resistance. In comparison, the addition of Fe accelerated the cathodic reaction, which is commonly termed as the rate-determining step for the degradation of biodegradable metals [52], and increased the *i*_corr_ value. Specifically, the ZnCuFe alloys exhibited a significantly increased *i*_corr_ value in α-MEM compared to ZnCu alloy (17.82 μA/cm^2^ for ZnCuFe (0.1 wt%), 16.78 μA/cm^2^ for ZnCuFe (0.2 wt%) and 16.43 μA/cm^2^ for ZnCuFe (0.4 wt%). A higher *i*_corr_ value was also observed for ZnCuFe alloys in artificial saliva (2.38 μA/cm^2^ for ZnCuFe (0.1 wt%), 3.02 μA/cm^2^ for ZnCuFe (0.2 wt%) and 5.29 μA/cm^2^ for ZnCuFe (0.4 wt%) compared to that for ZnCu alloy. Note that the *i*_corr_ value of ZnCuFe alloy apparently enhanced with increasing Fe content in artificial saliva, which was not observed in α-MEM.

**Table 1:** The fitting results of PDP and EIS data.

Electrolyte	Sample	Polarization fitting		EIS fitting						
		*E*_corr_ (V_SCE_)	*i*_corr_ (μA cm^−2^)	R_s_ (Ω cm^2^)	Q_p_*10^−7^ (s^n^ Ω cm^−2^)	n_1_	R_p_ (Ω cm^2^)	Q_ct_*10^−6^ (s^n^ Ω^−1^ cm^−2^)	n_2_	R_ct_ (kΩ cm^2^)
**α-MEM**	Zn	−0.89	14.67	11.21	2.49	0.87	13.36	10.42	0.89	1.59
	ZnCu	−1.20	10.68	20.44	2.11	0.92	34.74	7.99	0.92	1.98
	ZnCuFe (0.1)	−1.12	17.82	7.28	71.43	0.91	15.49	0.06	0.99	1.26
	ZnCuFe (0.2)	−0.92	16.78	13.16	7.57	0.89	8.96	8.49	0.91	1.08
	ZnCuFe (0.4)	−0.93	16.43	16.63	1.41	0.91	8.93	6.41	0.90	1.12
**Artificial saliva**	Zn	−0.93	2.68	80.42	14.52	0.98	248.54	19.57	0.56	2.71
	ZnCu	−0.94	1.99	46.77	0.39	0.99	471.63	4.63	0.82	3.44
	ZnCuFe (0.1)	−0.87	2.38	68.26	12.69	0.97	383.98	6.64	0.75	2.52
	ZnCuFe (0.2)	−0.89	3.02	45.96	0.92	0.98	330.75	2.86	0.86	1.92
	ZnCuFe (0.4)	−0.91	5.29	50.59	131.26	0.63	373.02	0.63	0.93	1.32

The EIS results showed a similar tendency as observed for PDP measurements. The ZnCu alloy showed the largest impedance loop among all the samples in both electrolytes. The addition of Fe reduced the impedance value remarkably. To decode the impedance data of samples quantitatively, the equivalent circuit is proposed as R_s_ (Q_p_ (R_p_ (Q_ct_R_ct_))), where R_s_ represents the resistance of electrolyte; Q_p_ and R_p_ refer to the capacitance and resistance of the product layer formed on the sample surface, respectively; and Q_ct_ and R_ct_ are the double-layer capacitance and resistance of the interfacial charge transfer reaction, respectively. Among the parameters, R_ct_ is commonly used as an important indicator to evaluate the overall corrosion reactions. It was found that the ZnCu alloy possessed the highest R_ct_ values (1.59 kΩ cm^2^ in α-MEM, 3.44 kΩ cm^2^ in artificial saliva) compared to other samples. The R_ct_ values for ZnCuFe (0.1 wt%), ZnCuFe (0.2 wt%) and ZnCuFe (0.4 wt%) in α-MEM were 1.26, 1.08 and 1.17 kΩ cm^2^, respectively, lower than that of ZnCu alloy. Note that ZnCuFe alloys in artificial saliva showed a remarkable decrease in R_ct_ with an increase of Fe concentration. The R_ct_ values for ZnCuFe alloys declined from 2.52 kΩ cm^2^ for ZnCuFe (0.1 wt%) to 1.92 kΩ cm^2^ for ZnCuFe (0.2 wt%) and further to 1.32 kΩ cm^2^ for ZnCuFe (0.4 wt%).

**Fig. 5:**
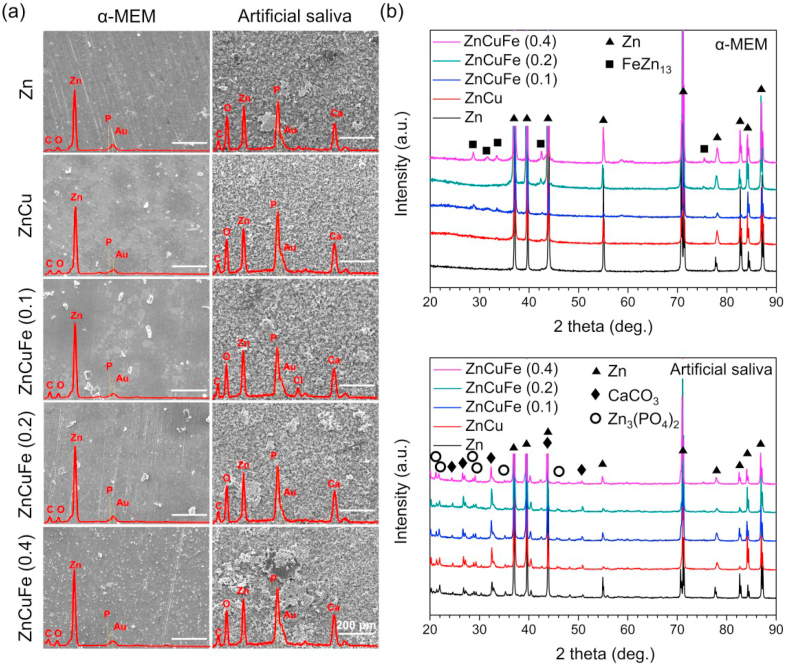
*In vitro* degradation behavior of pure Zn and the investigated Zn-based alloys. (a) Representative SEM images of surface morphology after 21 days of immersion in α-MEM and artificial saliva at 37 ± 0.5 °C; the EDS measurement shows the whole area detected and the corresponding spectra (inset red line) indicate the elemental composition of the degradation products (energy range between 0 and 5 keV). (b) XRD patterns of Zn, ZnCu, ZnCuFe (0.1 wt%), ZnCuFe (0.2 wt%) and ZnCuFe (0.4 wt%) after 21 days of immersion in α-MEM and artificial saliva at 37 ± 0.5 °C; The phase Zn (reference card 00-004-0831), FeZn_13_ (reference card 03-065-1238), CaCO_3_ (reference card 01-086-2341), Zn_3_(PO_4_)_2_ (reference card 00-011-0035) were identified.

Fig. 5a depicts the surface morphology and elemental composition of the samples after 21 days of immersion in α-MEM and artificial saliva. The corrosion products were also determined by XRD (Fig. 5b). The surface morphology of samples after immersion in α-MEM was similar. No obvious corrosion product layer was observed. Instead, a small amount of degradation particles could be found distributed over the surfaces. Note that a denser distribution of degradation particles with smaller size was observed on ZnCuFe alloys with increasing Fe content. EDS analysis indicated that the surfaces were mainly composed of Zn, C, O and a small amount of P. The ZnCuFe (0.4 wt%) showed a much higher P element peak compared to other samples. Whereas, only Zn and FeZn_13_ phase were detected by XRD, mainly due to the limited amount of corrosion products. On the contrary, all the samples in artificial saliva were entirely covered by the degradation product layers. Among these, the layer formed on ZnCu alloy was homogeneous. After alloying with Fe, partially accumulated degradation products appeared and their number increased with increasing Fe content. According to the EDS analysis, the presence of C, O, Zn, P and Ca was detected on the surface. The XRD analysis revealed that the main corrosion products were CaCO_3_ and Zn_3_(PO_4_)_2_.

**Fig. 6:**
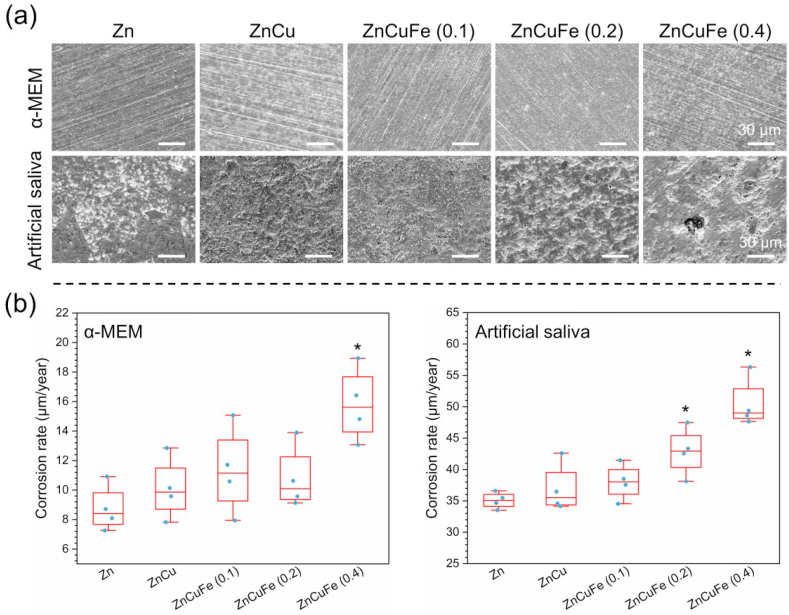
(a) Representative corrosion morphology after removing the corrosion products and (b) the corrosion rate calculated by weight loss of Zn, ZnCu, ZnCuFe (0.1 wt%), ZnCuFe (0.2 wt%) and ZnCuFe (0.4 wt%) after 21 days of immersion in α-MEM and artificial saliva at 37 ± 0.5 °C. * represents p < 0.05 when compared to pure Zn.

The corrosion products were removed to further observe the corrosion underneath the surface layer of samples (Fig. 6a). All the samples in α-MEM exhibited a similar corrosion morphology after 21 days of immersion. The grinding scratches could be still observed, indicating mild aggression to Zn-based materials in the α-MEM medium. In contrast, the substrates of samples were severely damaged in the artificial saliva. Specifically, ZnCu, ZnCuFe (0.1 wt%) and ZnCuFe (0.2 wt%) exhibited a relatively uniform corrosion morphology, similar to that of pure Zn. Nevertheless, a tendency towards severe localized corrosion was observed with increasing Fe content over 0.2%. Distinct corrosion pits were found on the surface of ZnCuFe (0.4). This tendency was further confirmed by the corrosion/degradation rate measurements (Fig. 6b). All the samples immersed in the artificial saliva showed a much higher degradation rate compared to the samples immersed in the α-MEM medium. Moreover, the degradation was significantly accelerated when the Fe content reached 0.2 wt% in the artificial saliva. The ZnCuFe (0.2 wt%) possessed a degradation rate of 42.4 μm/year, significantly higher than that of pure Zn (35.1 μm/year). Furthermore, the degradation rate of ZnCuFe (0.4 wt%) further increased to 50.4 μm/year. Nonetheless, in the α-MEM medium the degradation acceleration on the ZnCuFe (0.1, 0.2 wt%) samples was not so significant as in the artificial saliva; and only the ZnCuFe (0.4 wt%) displayed an apparently accelerated degradation rate (16.0 μm/year), compared to that of pure Zn (8.7 μm/year).

### 3.3 Cytotoxicity evaluation

**Fig. 7:**
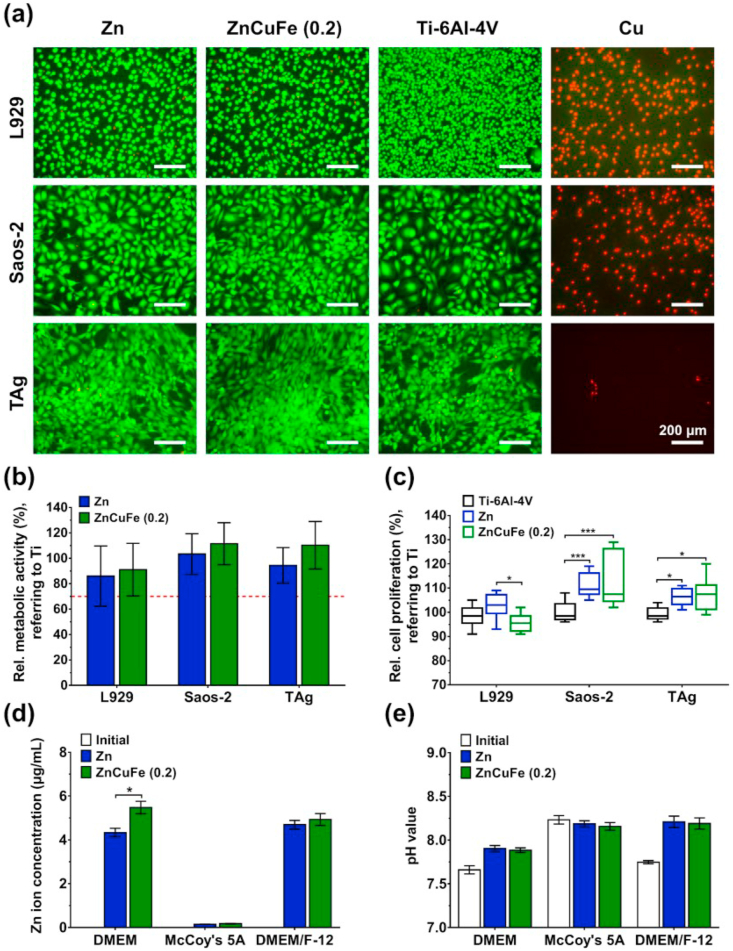
Cytotoxicity assessment of pure Zn and ZnCuFe (0.2 wt%) alloy. (a) Live/dead fluorescent images of L929, Saos-2 and TAg cells cultured in sample extracts for 24 h (magnification 100×, scale bar = 200 μm). Ti–6Al–4V extracts were used as negative controls, and pure Cu as positive controls. (b) Relative cell metabolic activities of L929, Saos-2 and TAg cells after 24 h of incubation with sample extracts determined by CCK-8 assay. Dashed line indicates 70% of control; relative metabolic activities below 70% are considered to show cytotoxic effects. (c) Relative cell proliferation of L929, Saos-2 and TAg cells after 24 h of incubation with sample extracts determined by BrdU assay; *p < 0.05, ***p < 0.001. (d) Zn ion concentration (μg/mL) determined by ICP-OES; *p < 0.05. (e) pH values of sample extracts.

Fig. 7a depicts the live/dead fluorescent images of L929, Saos-2 and TAg cells after exposure to sample extracts. Each cell line cultured in ZnCuFe (0.2 wt%) extracts exhibited good cellular responses with spindle-shaped attachment and showing partially pronounced cell-to-cell connections, comparable with those of negative controls cultured without extracts. Most stained cells showed green fluorescence, implying cell membrane integrity. To determine cell viability quantitatively, the relative metabolic activity was measured by CCK-8 assay, as shown in Fig. 7b. All cells cultured in ZnCuFe (0.2 wt%) extracts displayed cell metabolic activities above 70% of the control, reflecting no cytotoxic impact of tested extracts. Additionally, no significant differences were observed between Zn and ZnCuFe (0.2 wt%) extracts (p > 0.05). Fig. 7c shows the relative cell proliferation of L929, Saos-2 and TAg cells cultured in the presence of extracts of pure Zn and ZnCuFe (0.2 wt%) alloy, determined by BrdU assay. No cells exposed to the sample extracts exhibited apparent inhibition of cell proliferation (> 70% of control). Relative proliferation of Saos-2 and TAg cells increased significantly in the presence of Zn and ZnCuFe (0.2 wt%) extracts compared to the negative control (p < 0.05). However, ZnCuFe (0.2 wt%) extracts induced a significant decrease in proliferation activities of L929 cells in comparison to pure Zn extracts (p < 0.05). As shown in Fig. 7d, only the Zn ion release could be detected out by the ICP-OES method for the samples. The other metallic ions (Cu and Fe) could not be measured (probably below the detection limit), which might be attributed to the very low contents of Cu and Fe as well as their lower probability of anodic dissolution compared to the Zn matrix. In DMEM medium, the concentration of Zn ions released from the ZnCuFe (0.2 wt%) alloys was significantly higher than that of pure Zn (p < 0.05). Regarding McCoy's 5A and DMEM/F-12 media, no significant differences in Zn ion concentration between pure Zn and ZnCuFe (0.2 wt%) extracts were observed (p > 0.05). In McCoy's 5A medium, the Zn ion concentration was lower (< 0.5 μg/mL) than that in DMEM or DMEM/F-12. This might be caused by the fact that McCoy's 5A contains a higher concentration of HPO_4_^2−^, leading to an increased formation of passivation film [45]. Except for McCoy's 5A medium, the pH values of Zn and ZnCuFe (0.2 wt%) extracts were increased compared with the controls, whereas all pH values were below 8.5.

### 3.4 Evaluation of antibacterial properties

**Fig. 8:**
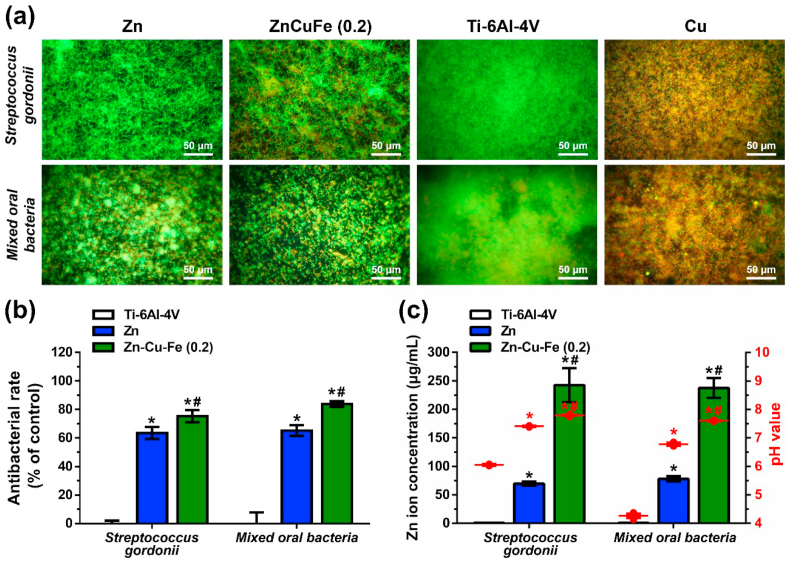
Antibacterial properties of pure Zn and ZnCuFe (0.2) alloy. (a) Bacterial adhesion and biofilm formation on Ti–6Al–4V alloy and selected ZnCuFe (0.2 wt%) alloy after incubation with *S. gordonii* and mixed oral bacteria for 12 h. Representative fluorescence images obtained by live/dead staining (magnification 400 × , scale bar = 50 μm). (b) Antibacterial rates of pure Zn and ZnCuFe (0.2 wt%) alloy to both planktonic *S. gordonii* and mixed oral bacteria after 12 h of incubation. Bacterial suspensions without samples were used as controls and normalized to 100%. (c) Zn ion concentrations (μg/mL) and pH values (red lettering) in collected bacterial media after culturing with tested samples for 12 h * and # represent p < 0.05 when compared to either Ti–6Al–4V or pure Zn, respectively.

The antibacterial properties of the ZnCuFe (0.2 wt%) alloy determined by live/dead staining are depicted in Fig. 8a. The Ti–6Al–4V surface showed an intense fluorescent staining after 12 h of incubation with *S. gordonii* and mixed oral bacteria, indicating the formation of dense films on this surface. Pure Cu surfaces showed red fluorescent staining after culturing with *S. gordonii* and mixed oral bacteria, indicating nonviable bacteria. In contrast, relatively fewer vital bacterial layers were found on the surfaces of ZnCuFe (0.2 wt%) and pure Zn inoculated with *S. gordonii*. Mixed oral bacteria which exhibited point-like fluorescent staining and much lower density (no coherent biofilm formation). This indicates that the ZnCuFe (0.2 wt%) surface inhibited partially the bacterial adhesion and biofilm growth of both *S. gordonii* and the multi-species oral biofilms after 12 h of incubation.

The antibacterial rate for planktonic bacteria was used to evaluate quantitatively the relative bacteria growth exposed to the tested sample after 12 h incubation, as shown in Fig. 8b. The pure Zn and ZnCuFe (0.2 wt%) alloy had an apparent inhibition to the planktonic bacterial growth rates of both *S. gordonii* and the multi-species oral bacteria when compared to those of Ti–6Al–4V (p < 0.05). Also, statistically significant differences between pure Zn and ZnCuFe (0.2 wt%) alloy were observed (p < 0.05), indicating the superior antibacterial properties of ZnCuFe (0.2 wt%) alloy compared to pure Zn. To further analyze the potential reason, metallic ion concentration and pH value in bacteria media were measured, as shown in Fig. 8c. According to ICP-OES measurements, Cu and Fe ion concentration in the bacteria media was hardly detectable due to the detection limit. Thereby, the ion changes in the respective bacterial media were mainly arisen from the Zn ion releasing. The amount of Zn ion release through ZnCuFe (0.2 wt%) alloys was almost three-fold higher than that of pure Zn (p < 0.05). This tendency could be observed in both *S. gordonii* media and mixed oral bacteria media. Furthermore, significant differences in pH values among the respective bacterial media were observed (Fig. 8c). Regarding the Ti–6Al–4V and control group, the bacterial media of *S. gordonii* and mixed oral bacteria showed acidic values (the range of pH values: 4.1–6.1). In contrast, the pure Zn and ZnCuFe (0.2 wt%) alloys cultured media tended to neutral or slightly alkaline values (the range of pH values: 6.8–7.8). Notably, the pH values of ZnCuFe (0.2 wt%) cultured media were higher than those of pure Zn cultured media (p < 0.05).

## 4 Discussion

An ideal material for GBR membranes ought to meet the following fundamental requirements:

(i) sufficient mechanical properties to provide a stable space for bone regeneration,
(ii) appropriate biodegradability to not only degrade and metabolize fully *in vivo* but also to well match new bone formation,
(iii) excellent biocompatibility so that there are no adverse effects on the surrounding tissue, and
(iv) potential antibacterial properties to reduce the risk of postoperative infections [1,2,[4], [5], [6]].

Therefore, we investigated the feasibility of using hot-extruded ZnCuFe alloys for applications as GBR membranes, especially for specific requirements at implantation sites.

### 4.1 Microstructure and mechanical properties of ZnCuFe alloys

Sufficient mechanical strength is an essential requirement to maintain suitable space and withstand surrounding forces. Ti membrane or Ti-reinforced membrane provides excellent mechanical strength for GBR treatment to meet the majority of clinical requirements, especially for vertical ridge augmentation [53]. A previous study reported that the mechanical properties of pure Zn membranes are inferior to those of pure Ti membrane [14]. Therefore, in this study, a series of hot-extruded ZnCuFe ternary alloys were fabricated and analyzed.

According to the binary phase diagram of Zn–Cu system [54], the low temperature solid phase (solid solution phase) is supposed to be formed directly from the liquid at approx. 419.5 °C when the Cu concentration is less than 1.7 wt%. Since no precipitate phase was observed in Zn–1.53 wt% Cu alloy during solidification [55], this indicates the high solid solubility of Cu in the Zn matrix at room temperature. Meanwhile, it has been reported that the Zn–1Cu alloys (wt%) showed no presence of precipitates after hot-extrusion [28]. Therefore, the secondary phase was not observed in the ZnCu alloy due to the low Cu concentration (0.5 wt%), and the presence of Cu demonstrated that it was dissolved in the Zn matrix. This was further confirmed by the elemental mapping analysis (Fig. S1) as the Cu element was uniformly distributed in the matrix and there was no Cu-containing secondary phase detected out. Moreover, after alloying with Fe, only FeZn_13_ secondary phase was found in the ZnCuFe alloys. It has been reported that a Zn–Cu–Fe alloy system can be simplified as Zn–Cu and Zn–Fe binary alloys for the analysis of solidification behavior [34]. Herein, the phase precipitation behavior of ZnCuFe alloys can be regarded as that of Zn–Fe alloy because of the complete dissolution of Cu element in the Zn matrix. According to the Zn–Fe binary phase diagram [56], FeZn_13_ precipitated directly from the liquid when the melt temperature was below 530 °C for the ZnCuFe (0.1 wt%) and ZnCuFe (0.2 wt%) alloys, whereas a peritectic reaction L + FeZn_10_ → FeZn_13_ occurred at 530 °C for the ZnCuFe (0.4 wt%) alloy. Thus, the FeZn_13_ precipitated phase was observed for all the ZnCuFe alloys. The volume fraction of FeZn_13_ phase increased remarkably when the Fe content increased from 0.1 to 0.2 wt%, whereas some FeZn_13_ phase particles were observed to have a diameter of more than 10 μm as the Fe content increased to 0.4 wt%. As reported, the diameter of the FeZn_13_ phase is more than 50 μm in Zn–1.3%Fe alloy [33]. Apparently, a higher Fe content in Zn-based alloys induces a significant increase in the size of the FeZn_13_ phase.

ZnCu alloy showed great tensile properties compared to pure Zn. Due to the solid solution strengthening, the tensile strength and ductility were both significantly enhanced. After alloying with Fe, the mechanical strength was further promoted. On the one hand, the uniformly distributed FeZn_13_ particles can hinder the movement of dislocations and thus increase the strength of the alloy. On the other hand, the grain size can be further refined by the presence of FeZn_13_ phase during the hot-extrusion process. It has been found that the deformation zone around the hard second phase during processing could accelerate the dynamic recrystallization process and refine the grains of Zn matrix [12]. However, the elongation at fracture of ZnCuFe (0.4 wt%) declined dramatically, even to much lower values than that of pure Zn. This is supposed to happen mainly due to the size of intermetallic particles. It is generally known that the deformation of metals is based on the movement of dislocations in the matrix. Particles with too large size can prevent dislocations passing, leading to a dislocation pile-up and the formation of cracks, finally resulting in a decrease in ductility [34]. Herein, the ZnCuFe (0.2 wt%) alloy showed a good balance between strength and ductility, with a UTS of 202.3 MPa and an elongation at fracture of 41.2%, comparable to those values of pure Ti membrane (Grade I) [14].

### 4.2 Biodegradability of ZnCuFe alloys

Regarding the degradation environment of GBR membranes, the main body fluid is human interstitial fluid owing to its location under the mucoperiosteum. Previous studies have demonstrated that *in vitro* degradation behavior under cell culture conditions might be comparable to that in *in vivo* physiological environments [57,58]. Notably, the most frequent postoperative complication is the GBR membrane exposure, with rates up to approximately 20% [59]. This indicates that the degradation environment switches to human saliva after exposure of GBR membrane to the oral cavity environment. Therefore, evaluating the degradation of Zn-based materials in cell culture medium and artificial saliva is essential for the application as suitable GBR membranes.

In our results, the ZnCu alloy showed the highest corrosion resistance among all the samples in both α-MEM and artificial saliva. Notably, the addition of Fe accelerated the degradation process according to the electrochemical results. This can be mainly attributed to the galvanic cell formed between FeZn_13_ phase and Zn matrix. The FeZn_13_ served as an active cathodic phase to Zn matrix, absorbing electrons produced from the oxidizing Zn matrix (anodic sites) [60]. Regarding degradation rate, the ZnCuFe (0.2 wt%) and ZnCuFe (0.4 wt%) showed 20.8% and 43.6% respectively increases relative to pure Zn in the artificial saliva, although in the α-MEM medium the acceleration is not so significant until the addition of Fe reaches 0.4 wt%. It was reported that the amino acids contained in α-MEM promoted the precipitation of phosphates after bounding to sample surfaces [61]. Accordingly, zinc phosphate tends to precipitate after higher Zn ion release from the anodic sites with increased Fe content in α-MEM, which was confirmed by the EDS results. The formed zinc phosphate can effectively inhibit the corrosion of the matrix [62], especially in such a mild α-MEM medium. Thus, the acceleration by Fe was set-back. In comparison, artificial saliva is more aggressive than α-MEM probably partially due to its much lower pH value (pH = 6), which tends even to dissolute the zinc phosphate precipitate. Hence, the fresh matrix was easily exposed under the attack of electrolytes and galvanic corrosion. Therefore, the higher Fe content induced a further breakdown of the passive layer and accelerated the degradation process. Moreover, with the extension of immersion time, a large amount of released Zn ions will participate in the formation of corrosion products (CaCO_3_ and Zn_3_(PO_4_)_2_) on the surface. Nonetheless, the galvanic corrosion underneath the surface layer is reported to be aggravated by incomplete coverage of the surface with a passive layer along with the existence of Cl^−^ [63,64]. Thus, the non-passivating corrosion product layer might in return facilitate galvanic corrosion underneath, between the secondary phase and the matrix, leading to an accelerated degradation process. However, when the Fe content increased to 0.4 wt%, severe localized corrosion was observed, which might cause a premature failure of the membrane. Taken together, the ZnCuFe (0.2 wt%) alloy shows in α-MEM and artificial saliva an improved degradation behavior and can be considered as a potential material for GBR applications.

It must be stressed that the long-term degradation test showed the ZnCuFe alloys degraded faster in the artificial saliva than in the α-MEM, which seems contradictory with the transient electrochemical corrosion result. Such phenomenon does occur to reactive metals because the long-term degradation profile depends on not only the initial thermodynamic state but also the evolving kinetic factors especially of the interplay between the electrolyte and metal surface. Note the compositions of the two test media are distinctly different as shown in Table S1. Although the artificial saliva is an aggressive electrolyte as mentioned above, it contains also the organic carbamide which has been reported to inhibit the dissolution of Zn in the acidic environment via physical adsorption [65]. Likewise, the artificial saliva tends to form more readily such an initial conditioning film on the surface than the α-MEM, which serves as a protective layer to prevent corrosion. Hence, the transient electrochemical corrosion showed the ZnCuFe alloys had a lower *i*_corr_ as well as a higher R_ct_ in the artificial saliva than in the α-MEM. However, this can occur only for a short term at early stage of the degradation. As the immersion time prolonged, the afore-mentioned dynamic factors could play a more important role in governing the alloy's degradation profile. For instance, the newly formed corrosion products may break the absorbed or conditioning film. Such non-passivating covering could facilitate the underneath corrosion as discussed above. Some inorganic constituents, such as thiocyanate ions (SCN^−^), of the artificial saliva, along with its low pH value, could exacerbate reportedly the localized corrosion during long-term immersion [66]. Thus, the ZnCuFe alloy tends to undergo a relatively high degradation rate in the artificial saliva in long-term immersion. However, this does not occur to the α-MEM. On the contrary, α-MEM contains some organic components such as l-glutamine albeit which plays reportedly an opposite role in influencing the Zn corrosion, i.e. it may attack the Zn surface and accelerate its dissolution by preventing the corrosion products formation [67]. Whereas, the l-glutamine is easily decomposed particularly when the pH value increases caused by the cathodic reaction of Zn corrosion [68]. For long-term immersion degradation of the alloy, other organic constituents such as amino acids could absorb on the surface and serve to protect the underneath Zn against corrosion. In short, for the biodegradable Zn-based alloys their transient electrochemical corrosion results could not be directly translated into its long-term degradation prediction which must be investigated independently.

### 4.3 Cytocompatibility of ZnCuFe alloy

Undoubtedly, biocompatibility is one of the prerequisite factors to be evaluated for a potential biomaterial. In this study, the hot-extruded ZnCuFe (0.2 wt%) alloy was chosen for biological evaluation due to its sufficient mechanical strength and appropriate degradation behavior. Herein, an indirect cytotoxicity test by extract assay was utilized to determine the effects of the degradation products released from ZnCuFe (0.2 wt%) alloy towards cells. Fibroblasts, osteoblast-like, and immortalized periosteal cells were used, as they represent surrounding cells for the clinical application as GBR membranes.

The results indicate that the hot-extruded ZnCuFe (0.2 wt%) alloy exhibits no apparent cytotoxic effects (Fig. 7a and b). In principle, *in vitro* cytotoxicity results are determined by the cellular tolerance of degradation products released from the ZnCuFe (0.2 wt%) alloys [69]. Analysis of the sample extracts revealed an increase in Zn ion concentration and pH values due to cathodic and reduction reactions in the cell culture medium (Fig. 7e and f). It has been reported that a pH value lower than 9 in culture medium has no adverse effects on the viability of L929 and TAg cells [21,70]. Additionally, previous studies confirmed that the cytotoxicity of Zn and its alloys is related to the Zn ion concentration in the extracts [45]. Our results also demonstrated that the range of Zn ion concentrations (< 5.4 μg/mL) in sample extracts were lower than the cellular tolerance of L929, TAg and Saos-2 cells, as previously measured [12,21]. Nevertheless, the released Zn ion concentration modulates the cellular responses, such as cell viability, proliferation and osteogenic differentiation [21,71]. As shown in Fig. 7c, a lower proliferation rate of L929 cells was found in the presence of ZnCuFe (0.2 wt%) extracts compared to that detected in pure Zn (p < 0.05). This might be caused by the higher Zn concentration released from the ZnCuFe (0.2 wt%) alloys (Fig. 7d), which might have been close to the cellular tolerance for Zn ion concentration. Similarly, the proliferation rates of Saos-2 and TAg cells were promoted by the specific Zn ion concentrations in the sample extracts (Fig. 7d). It must be emphasized that according to ISO standard 10993-5 and −12, a combination of indirect and direct contact test is recommended to screen the potential cytotoxic effects of bioinert materials. Nonetheless, for biodegradable metals such as Zn alloys the *in vitro* direct cultivation of cells on the surface might be hampered by the rapid degradation and partly shedding of degradation products. Thereby, the indirect cytotoxicity test by extract assay is preferred for initial screening in most current studies of Zn-based alloys. Based on our results, the hot-extruded ZnCuFe (0.2 wt%) alloys reveal an appropriate cytocompatibility, which was mainly determined by the concentration of the released Zn ions.

### 4.4 Antibacterial properties of ZnCuFe alloy

In fact, occurring infections after GBR membrane exposure is a common complication in the postoperative period, with rates of up to 20% for non‐biodegradable membranes and 5% for biodegradable ones [72]. Generally, the antibacterial properties of biomaterials ought to be effective against infection-related microorganisms. Due to a transoral surgical approach, postoperative oral infections usually involve multiple species of bacteria, including streptococci, anaerobic Gram-negative rods and anaerobic Gram-positive cocci [47,73]. *S. gordonii* plays a critical role in initial colonization through the formation of biofilm in the oral environment to which other colonizers can adhere [46,74]. In the present study, the antibacterial properties of ZnCuFe (0.2 wt%) alloys were investigated after exposure to *S. gordonii* and mixed oral bacteria which may colonialize membranes during GBR applications in the oral cavity. Our results showed that ZnCuFe (0.2 wt%) surfaces possess the ability to inhibit the initial adhesion of *S. gordonii* and mixed oral bacteria compared with Ti–6Al–4V surfaces (Fig. 8a). Based on the quantitative results, the ZnCuFe (0.2 wt%) alloys led to higher antibacterial rates compared to those of pure Zn (Fig. 8b), probably attributed to increased released Zn ions and an alkaline shift in pH values (Fig. 8c).

Previous studies have also demonstrated that Zn–Cu alloys showed good antibacterial abilities towards *Staphylococcus aureus* (*S. aureus*) [28,29,75]. The detailed antibacterial mechanisms of Zn–Cu alloys is not completely understood but it is probably linked to the release of metallic ions (Zn^2+^ and Cu^2+^) and hydroxide ions (which increases the pH value). A previous study reported that Zn ions are considered to elicit effective bacteriostatic effects towards *S. aureus*, rather than to possess bactericidal effects [76]. Due to the different cell wall structures and the related Zn ion transport mechanisms, the Gram-positive bacterium *S. aureus* is more tolerant to the antibacterial effects of released Zn ions than the Gram-negative bacterium *Escherichia coli* [77]. This might explain that the ZnCuFe (0.2 wt%) surface only exhibits an inhibitory effect on *S. gordonii* (Gram-positive) and mixed oral bacteria. Cu-containing metallic biomaterials have been considered to possess effective antibacterial properties [78,79]. A previous study demonstrated that the antibacterial properties can be enhanced by increasing the Cu content in Zn–Cu alloys [28]. Nonetheless, in our study the Cu ions released from the ZnCuFe (0.2 wt%) alloys, which are below the detection limit, might play only a minor role in the anti-bacterial performance. Whereas, the undissolved Cu contained in the matrix may play a part albeit this remains unclear. In turn, the alloying elements in particular the Fe promoted significantly the release of Zn ions on the ZnCuFe (0.2 wt%) alloy (Fig. 8c), caused by the galvanic corrosion between the Zn matrix and the secondary phase. Thereby, the enhanced Zn dissolution occurred concomitantly with the significantly higher antibacterial rates on the ZnCuFe (0.2 wt%) alloy, and verifies the main role of Zn ion played in its anti-bacterial effect (Fig. 8b). Additionally, a potential explanation for antibacterial properties might be that the cathodic reaction (releasing hydroxide ions) increases the pH value in the medium, which was also verified by our measurements (Fig. 8c). The apparent alkaline shift in pH values could be observed in bacterial media of pure Zn and ZnCuFe (0.2 wt%) alloys, probably due to the absence of buffering agents in the Schaedler medium. It has been reported that Mg-based alloys exhibited antimicrobial properties *in vitro,* mainly due to their rapid degradation, leading to an increase in the pH value [80]. Nevertheless, the antibacterial effect is obviously diminished *in vivo* because of the buffering effect of body fluids. Although the antibacterial properties of the ZnCuFe (0.2 wt%) alloys can be attributed to multiple effects, the exact antibacterial mechanism ought to be determined by a more in-depth examination.

## 5 Conclusion

In the present work, newly developed Zn–0.5Cu–xFe (x = 0.1, 0.2 and 0.4 wt%) alloys were investigated as potential materials for the fabrication of GBR membranes. The principal conclusions are as follows:

1. The FeZn_13_ phase was the solely formed secondary phase after the addition of Fe to ZnCu alloy. Although increase of Fe content tends to reduce the elongation at fracture of Zn–0.5Cu–xFe alloys due to the increasing size of the secondary phase, the ZnCuFe (0.2 wt%) achieved still a UTS of 202.3 MPa and elongation at fracture of 41.2%, comparable to those of pure Ti membranes.
2. The addition of Fe accelerated the degradation of the formed Zn–0.5Cu–xFe alloys in both α-MEM medium and artificial saliva, which was mainly contributed to the galvanic corrosion. The acceleration was set-back slightly in α-MEM due to the protection by formed zinc phosphate. The ZnCuFe (0.2 wt%) showed significantly enhanced and more uniform degradation behavior compared to pure Zn.
3. The extracts of ZnCuFe (0.2 wt%) alloys showed no cytotoxic effects towards L929 fibroblasts, Saos-2 osteoblasts or TAg periosteal cells, mainly due to released Zn ion concentration below the respective cellular tolerance. Moreover, the ZnCuFe (0.2 wt%) alloy has potential antibacterial effects, attributed mostly to the released Zn ions and the partly to the alkaline shift of the pH values.

Taken together, the ZnCuFe (0.2 wt%) alloy may become a new option as a suitable membrane material for GBR applications due to its optimized mechanical properties, improved degradation behavior, excellent cytocompatibility and antibacterial properties.

## CRediT authorship contribution statement

**Wentai Zhang:** Methodology, Formal analysis, Visualization, Writing - original draft. **Ping Li:** Methodology, Formal analysis, Visualization, Writing - original draft. **Gang Shen:** Investigation, Data curation. **Xiaoshan Mo:** Investigation, Data curation. **Chao Zhou:** Validation, Resources. **Dorothea Alexander:** Writing - review & editing. **Frank Rupp:** Writing - review & editing. **Jürgen Geis-Gerstorfer:** Conceptualization, Supervision, Funding acquisition, Resources. **Haijun Zhang:** Conceptualization, Supervision, Funding acquisition, Resources. **Guojiang Wan:** Conceptualization, Supervision, Funding acquisition, Resources.

## Declaration of competing interest

None.
