# Supplementary material for: Appropriately adapted properties of hot-extruded Zn–0.5Cu–xFe alloys aimed for biodegradable guided bone regeneration membrane application
Source: Bioact Mater. 2020 Oct 9;6(4):975–89. doi: 10.1016/j.bioactmat.2020.09.019 (PMC7560602; doi:10.1016/j.bioactmat.2020.09.019)
Supplement: Multimedia component 1 [file mmc1.docx]

**Table 1.** The fitting results of PDP and EIS data
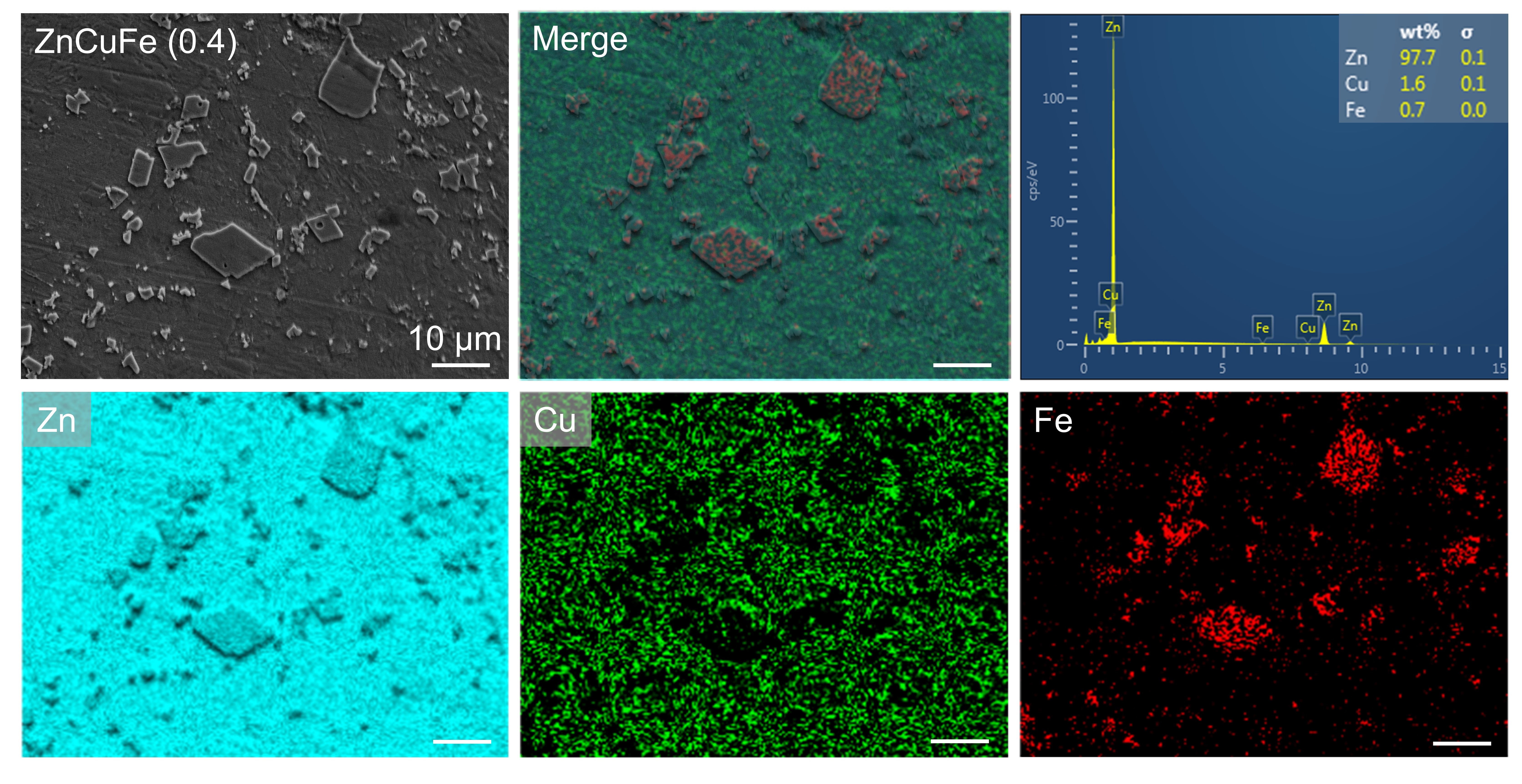


**Fig. S1.** Representative SEM images and corresponding elemental mappings of ZnCuFe (0.4 wt%) alloys.

| Composition | Inorganic ions (mM) | | | | | | | |  | Organic components (g/L) | | |
| --- | --- | --- | --- | --- | --- | --- | --- | --- | --- | --- | --- | --- |
|  | Na^+^ | K^+^ | Mg^2+^ | Ca^2+^ | Cl^-^ | SO_4_^2-^ | H_2_PO_4_^-^ | SCN^-^ |  | Glucose | Amino acids | Carbamide |
| Artificial saliva | 11.32 | 8.46 | - | 7.16 | 26.53 | - | 4.42 | 3.09 |  | - | - | 1.00 |
| α-MEM | 117.36 | 5.37 | 0.81 | 1.80 | 125.32 | 0.81 | 1.01 | - |  | 1.00 | 1.27 | - |

**Table S1.** Main compositions of artificial saliva and α-MEM
